# Heterologous overexpression, purification and functional analysis of plant cellulose synthase from green bamboo

**DOI:** 10.1186/s13007-019-0466-0

**Published:** 2019-07-25

**Authors:** Hsuan-Yu Huang, Yi-Sheng Cheng

**Affiliations:** 1 Institute of Plant Biology, National Taiwan University, Taipei, 10617 Taiwan; 2 Department of Life Science, National Taiwan University, Taipei, 10617 Taiwan; 3 Genome and Systems Biology Degree Program, National Taiwan University, Taipei, 10617 Taiwan

**Keywords:** CesA protein, Glycosyltransferase, Membrane protein purification, Cellulose microfibril, Cellulose synthase complex

## Abstract

**Background:**

The cellulose synthase complex (CSC), composed of cellulose synthase (CesA) proteins, is a catalytic enzyme complex involved in cellulose synthesis in the plant cell. CesA proteins synthesize cellulose microfibrils corresponding to the microtubule direction and export linear products across the plasma membrane. However, the CSC arrangement and the mechanism of cellulose synthesis in plant cells remain unclear. Purified CesA proteins are required to determine biochemical and biophysical characteristics.

**Results:**

In this study, we constructed, expressed, and purified six heterologously expressed cellulose synthases from *Bambusa oldhamii* (BoCesA) and analyzed the associated enzyme activity. The conjugating sequences of the maltose-binding protein (MBP) gene and the BoCesA genes were constructed into the expression vector pYES2/CT and were further transformed into yeast cells (BCY123) for fermentation culturing. Purified BoCesA recombinant proteins were obtained by a two-step purification procedure, consisting of immobilized metal affinity chromatography to purify MBP-BoCesAs and size-exclusion chromatography (Superdex-200) to isolate BoCesAs in oligomeric form. The enzymatic activity of oligomeric BoCesAs with 80% purity was determined by partially methylated alditol acetate (PMAA)-coupled gas chromatography–mass spectrometry (GC–MS) analysis. Furthermore, the long fiber-like products synthesized by oligomeric BoCesAs were observed under a transmission electron microscope (TEM) and were further confirmed as cellulose microfibril products.

**Conclusions:**

In this study, we successfully established a heterologous expression and purification system for BoCesAs. The purified recombinant BoCesA proteins display enzyme activity and can produce protein in milligram quantities for further studies on molecular composition and structure.

**Electronic supplementary material:**

The online version of this article (10.1186/s13007-019-0466-0) contains supplementary material, which is available to authorized users.

## Background

Cellulose, a major renewable substance derived from plant cell walls, is ubiquitous in woody plants. As an extensive feedstock for daily use, cellulose is an important source of bioethanol, thermal energy, and pulp production. Cellulose content could reach 40% in dicot woody plants [1], providing a stable source for pulp production [2]. With respect to fast-growing woody monocots, 3–5-year-old bamboo could be a potential polysaccharide resource for renewable material based on biomass yield [3] and composition analysis [4]. Several studies on fast-growing monocots have referenced the growth and development of moso bamboo, *Phyllostachys edulis,* demonstrating the mechanism of rapid shoot growth, cell wall biosynthesis and cell metabolism using draft genome sequencing [5], transcriptome sequencing [6] and signal pathway analysis [7].

Plant cell walls are composed of cellulose, hemicellulose and lignin [8]; cellulose is the major representative in the plant cell wall of a β-1,4 glucan with potential value. Cellulose microfibrils, the main load-bearing polymers of the plant cell wall, have specific inter-/intra-molecular hydrogen bonds and are important determinants of the physical characteristics of cell walls [9]. The cellulose synthase complex (CSC) is a transmembrane protein complex that synthesizes cellulose microfibrils in plant cells. The catalytic units in CSC, cellulose synthase (CesA) proteins, play the most important role in producing massive amounts of glucan. During cellulose synthesis, CesA proteins are guided by microtubules [10] to transfer glucosides of UDP-glucose into β-1,4 glucan chains [11] and to translocate these glucan chains to the cell wall to form cellulose microfibrils [12]. Catalyzed by the CesA protein in CSC, the cellulose is finally polymerized and exported to form the essential part of the plant cell wall.

Cellulose synthase complexes, which have been found in green algae, mosses and higher plants [13], were initially regarded as a cellulose microfibril-linked terminal complex [14] and have also been proposed as a rosette-like complex [15]. In the plasma membrane, CSC forms a 25-nm hexagonal rosette shape through assembly by different types of cellulose synthases [13]. In maize, a 36-CesA structure was proposed based on cellulose microfibril diameter analysis [16], i.e., the 36-chain cellulose microfibril cross-section could have the same arrangement of CesA proteins in CSC. In mosses, an 18-CesA structure was proposed by computational analysis [17], i.e., the CesA protein model could fit into the images of the rosette-like complex in the plasma membrane.

As indicated by sequence comparison, plant CesA homologs contain several conserved domains. The glycosyltransferase domain (GT-2 fold) with specific motifs (DD, DxD, ED, QxxRW) [17] in CesA proteins is a large catalytic domain that synthesizes cellulose using UDP glucose as substrate. The hydrophobic transmembrane helices provide both membrane anchoring and product translocation during cellulose biosynthesis [18]. In contrast to prokaryotic cellulose synthases [17, 19–21], in plant cellulose synthase the zinc-finger domain, hypervariable region, plant-conserved region and class-specific region are four specific insertions that might be involved in protein–protein interactions during CSC formation and movement [17, 19, 20].

In *Arabidopsis*, 10 cellulose synthase genes (AtCesA1–10) have been identified as playing a role in primary and secondary cell wall synthesis [22]. In general, genetic complementation and co-immunoprecipitation experiments have shown that cellulose biosynthesis in *Arabidopsis* cells involves up to three distinct AtCesAs [23, 24]. A series of research have indicated that AtCesA1, 3, 6 are involved in primary cell wall synthesis [25], AtCesA4, 7, 8 participate in secondary cell wall synthesis [26, 27] and AtCesA2, 5 show redundancy with AtCesA6 in primary cell wall synthesis [25]. In the primary cell wall of green bamboo, *Bambusa oldhamii*, 10 cellulose synthase genes (BoCesA 1–10) have been identified via gene structure and in situ gene expression analysis [28]. Phylogenetic analysis of the CesA amino acid sequences indicated that the BoCesAs associated with the primary cell wall are divided into three clades that correspond to the AtCesAs in *Arabidopsis*: BoCesA1, 2, 3, 8 and AtCesA1, 10 belong to Clade VI; BoCesA4, 5 and AtCesA3 belong to Clade V; and BoCesA6, 7 and AtCesA2, 5, 6, 9 belong to Clade I [28].

In this study, we establish a procedure for the milligram-scale purification of six cellulose synthases (BoCesAs) with detection of catalytic activity from *B. oldhamii* [28], which participate in primary cell wall formation. The total content of the target protein was improved by maltose-binding protein (MBP) conjugation and fermenter incubation. The recombinant CesA protein (MBP-BoCesA) and full-length CesA protein (BoCesA) were purified by immobilized metal affinity chromatography (IMAC) and size-exclusion chromatography (SEC), respectively. The BoCesA protein particles and the synthesized glucans could be observed using TEM, and the synthesized glucans were identified as β-1,4 glucans by linkage analysis. Full-length BoCesA proteins with high yield and enzymatic activity were obtained through this purification protocol.

## Experimental procedures and methods

### CesA genes, vector construction, and small-scale protein expression

The cDNA sequences of plant cellulose synthase genes from green bamboo (*B. oldhamii*) were provided by Dr. Ai-Yu Wang [28]; six full-length cellulose synthase genes were available. The DNA sequences of *BoCesA1*, *2*, *3*, *4*, *5*, *7* (Accession number: DQ020208, DQ020209, DQ020216, DQ020212, DQ020213, and DQ020215, respectively) were chosen for protein purification and functional assays.

For vector construction and small-scale protein expression, the *BoCesA5* gene was cloned into the expression vectors pET30a (Novagen) and pYES2/CT (Invitrogen) with and without an N-terminus MBP fusion tag. Briefly, expression vectors containing *BoCesA5*/*MBP-BoCesA5* sequences were transformed into *Escherichia coli* (strains BL21(DE3), C43, and RIL) and *Saccharomyces cerevisiae* (strain BCY123) for protein expression. The prokaryotic-grown BoCesA5 was expressed in LB broth (Neogen) medium via 8 h induction with 0.1 mM isopropyl β-d-1-thiogalactopyranoside (IPTG, Bionovas biotech) at 37 °C. The eukaryotic-grown BoCesA5/MBP-BoCesA5 was expressed in YPG medium (1% yeast extract, 2% peptone, and 2% galactose) and induced for 24 h at 30 °C. The cell pellets were collected at 4 °C for cell lysis and immunoblotting.

**Fig. 1:**
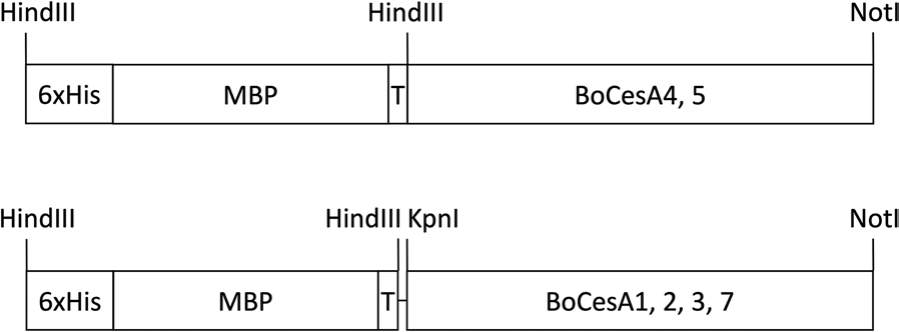
Open reading frame construction within the expression vector. Recombinant MBP-BoCesA and its related cloning site. *MBP* maltose-binding protein, *T* TEV protease cleavage site

For large-scale protein expression, *BoCesA* genes were first cloned into the pYES2/CT vector with different restriction enzyme sites (*BoCesA1*, *2*, *3*, *7*: *Kpn*I and *Not*I; *BoCesA4*, *5*: *Hin*dIII and *Not*I). A sequence insertion (6×His-MBP-cleavage site [TEV]) was subsequently cloned into the N-terminal site of BoCesA by *Hin*dIII site insertion which generated an MBP-BoCesA open reading frame (Fig. 1).

### Large-scale protein expression by fermentation culture

**Fig. 2:**
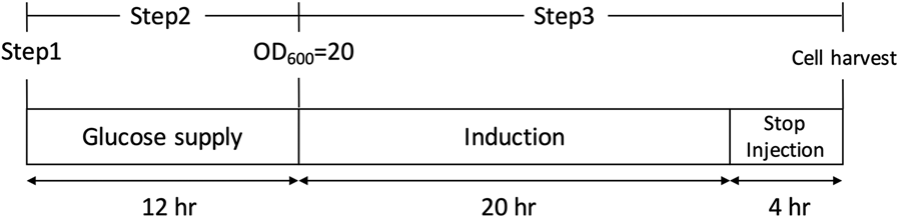
Fed-batch culture procedure. The process of three-step fed-batch culture. Step 1: Inoculation of yeast liquid culture; Step 2: Yeast cell culture with glucose; Step 3: Protein induction by galactose

The recombinant protein yield was improved by fermentation culture. Cell density in the fermenter (FM-01 and FS-V-D, Major Science) was 2.5-fold greater than that of flask culture for the same volume. Yeast were grown at 30 °C/pH 5.0 in sterile culture media with injection of filtered air to create positive pressure in the tank. The fermentation culture utilized a three-step expression procedure (Fig. 2): preparation (step 1), growing (step 2), and induction (step 3).

Step 1 consisted of preparation of the main medium of the tank by combining 60 g yeast extract, 120 g peptone, 20 g glucose, 36 g NaH_2_PO_4_, 6 g MgSO_4_·7H_2_O, and 5.5 L RO water with sterilization.

Step 2 consisted of inoculation of the medium in the tank with 250 mL high-density cell culture (OD_600_ > 30) and growth of yeast for 12 h with glucose supply (10% glucose, 400 mL). Cell density increased to OD_600_ = 20 after 12 h, at which point it was ready for induction.

Step 3 consisted of induction of the recombinant protein expression. Sterile fed-batch medium (50 g yeast extract, 100 g peptone, 4 g MgSO_4_·7H_2_O, 150 g galactose, and 2.1 L RO water) was injected into the tank within 20 h and cultured for an additional 4 h. The fermenter culture was then harvested (about 120 g in wet weight) and frozen until lysis.

### Primary purification of MBP-BoCesA protein

To prevent protein degradation, all purification procedures were performed at 4 °C. The frozen cells (about 50 g) were resuspended with 250 mL lysis buffer (20 mM NaH_2_PO_4_ pH8.0, 1 M NaCl, and 1 mM *tris* (2-carboxyethyl) phosphine (TCEP)) and mechanically lysed using a French press (TS2, Constant System Limited) at 35 kpsi. The debris was removed by centrifugation (9800×*g*, JA-14, J2-MC; Beckman) for 20 min and supernatant was collected for ultracentrifugation. The membrane pellet was harvested by ultracentrifugation (140,000×*g*, P28S, CP80WX, Hitachi) for 2 h and resuspended (about 8.5 g) by stirring in 100 mL resuspension buffer [20 mM NaH_2_PO_4_ pH 8.0, 1 M NaCl, 1 mM TCEP, 10% glycerol, 20 mM imidazole, and 0.5% *n*-dodecyl-β-d-maltopyranoside (DDM)] overnight.

After resuspension, the debris was removed by centrifugation (22,000×*g*, JA-20, J2-MC; Beckman) and the solution was filtered for purification. The MBP-BoCesA protein was purified using fast performance liquid chromatography (FPLC; ÄKTA Prime, GE Healthcare) on an immobilized metal affinity chromatography column (His-Trap, GE Healthcare). The membrane solution was injected into the column, washed with binding buffer (20 mM NaH_2_PO_4_ pH8.0, 1 M NaCl, 1 mM TCEP, 10% glycerol, 20 mM imidazole, and 0.05% DDM) and eluted with elution buffer (20 mM NaH_2_PO_4_ pH8.0, 1 M NaCl, 1 mM TCEP, 10% glycerol, 250 mM imidazole, and 0.05% DDM) to recover the MBP-BoCesA protein. The MBP-BoCesA protein was purified by recycling the membrane solution through a series of columns in order to increase protein yield. The recovered MBP-BoCesA protein was concentrated by centrifugal filtration (Amicon ultra-15 centrifugal filter, Merck Millipore) to about 1.7 mg/mL (10 µM) for secondary purification.

### Secondary purification of BoCesA protein

Secondary purification was performed to eliminate the fusion tag, recover BoCesA proteins, and determine their molecular mass. The MBP fusion tag was removed by adding a homemade TEV protease [29] with a concentration of 1.5 mg/mL. After a 12 h reaction at 4 °C, the digested sample was injected into a size-exclusion chromatography column (Superdex-200, GE Healthcare) using FPLC with gel-filtration buffer (20 mM NaH_2_PO_4_ pH 7.0, 0.3 M NaCl, 1 mM TCEP, 10% glycerol, and 0.005% FC-16) and UV absorbance (280 nm) monitoring. Ratios of molecular mass to column volume were calculated by regression analysis with gel filtration standards (Bio-Rad, 1511901). The purified BoCesA was concentrated by centrifugal filter to about 1.2 mg/mL (10 µM) and frozen at − 80 °C.

### Western blot and protein concentration

Protein samples were separated on an 8% SDS-PAGE gel, transferred to a polyvinylidene difluoride (PVDF) membrane, and hybridized with the Anti-BoCesA1/2/3 polyclonal antibody (against the HVR region of BoCesA8, for BoCesA1, 2, 3 detection), Anti-BoCesA4/5 polyclonal antibody (against HVR region of BoCesA5, for BoCesA4, 5 detection) [30], and maltose-binding protein antibody (GeneTex). The primary antibody-hybridized blot was incubated with horseradish peroxidase-conjugated secondary antibody. Chemiluminescent signals generated by Clarity Western ECL Substrate (Bio-Rad) were detected using UVP ChemStudio PLUS (Analytik Jena). The concentration of MBP-BoCesA and BoCesA proteins was determined by spectrophotometer (DS-11, Denovix).

### Determination of CesA enzymatic activity

Cellulose synthesis was monitored for 3 h at 25 °C in a 1 ml Eppendorf tube containing the reaction buffer [20 mM NaH_2_PO_4_ pH 5.5, 0.1 M NaCl, 1 mM MnCl_2_ and 0.005% Fos-Choline-16 (FC-16)], 0.5 µM (0.06 mg/mL) BoCesA protein, and 1 mM UDP-Glc. For amylase treatment, 0.1 U of amylase (Sigma, 10065) was added for 1 h at 25 °C. For cellulase treatment, 0.01 U of cellulose (Sigma, C1184) was added for 15 h at 25 °C. We subjected the synthesized cellulose to cellulase treatment and then observed the product by TEM to determine the synthesized microfibrils. For glucan analysis, cellulose synthesis, cellulase treatment, and amylase treatment were selected for preparing the glucan derivatives (partially methylated alditol acetates).

### Observation of protein particle/cellulose by transmission electron microscopy (TEM)

For the observation of protein particles, the purified BoCesA was diluted to 0.06 mg/mL. For the synthesized cellulose, the 1 mL samples were first centrifuged at 10,000×*g* at 4 °C for 30 min and the upper 900 µL solution was discarded. 5 µL sample solution from the remaining liquid was dropped onto a discharged carbon film-coated 300 mesh copper grid (CF300-Cu, Electron Microscopy Sciences) and stained with 2% uranyl acetate. The TEM images were taken by a Hitachi H-7650 TEM (75 kV, 30–120 K magnification, Erlangshen ES500W Model 782 CCD camera).

### Glucan linkage analysis

The 1 mL sample of solution was transferred to a 15 mL glass tube with 1 mL ethanol added and capped with a teflon-lined screw cap, then dried by a rotary evaporator (N-1000, Eyela). The remaining pellet was methylated, hydrolyzed, reduced, and acetylated following an existing protocol [31] for partially methylated alditol acetates (PMAA) preparation. The glucan derivatives for glucan linkage identification were injected into GC–MS (Agilent 5975/7890A GC) by heating in the HP-5MS fused silica capillary column (30 m × 0.25 mm × 25 µm, Agilent Technologies) at an injector temperature of 250 °C.

The helium carrier gas was operated at a constant flow rate of 1 mL/min. The oven was programmed as follows: 100 °C for 1 min, heating at a rate of 10 °C/min to 200 °C, 200 °C for 1 min, and heating at a rate of 15 °C/min to 300 °C.

## Results

### Establishing the purification platform via eukaryotic expression and tag conjugation

To express various CesA proteins, we chose CesA genes in the primary cell wall of *B. oldhamii* to establish the purification protocol. Based on amino acid sequence clustering [28], 6 BoCesA genes from 3 clades, Clade VI (*BoCesA1*, *2*, *3*), Clade V (*BoCesA4*, *5*) and Clade I (*BoCesA7*) were selected in this study.

**Fig. 3:**
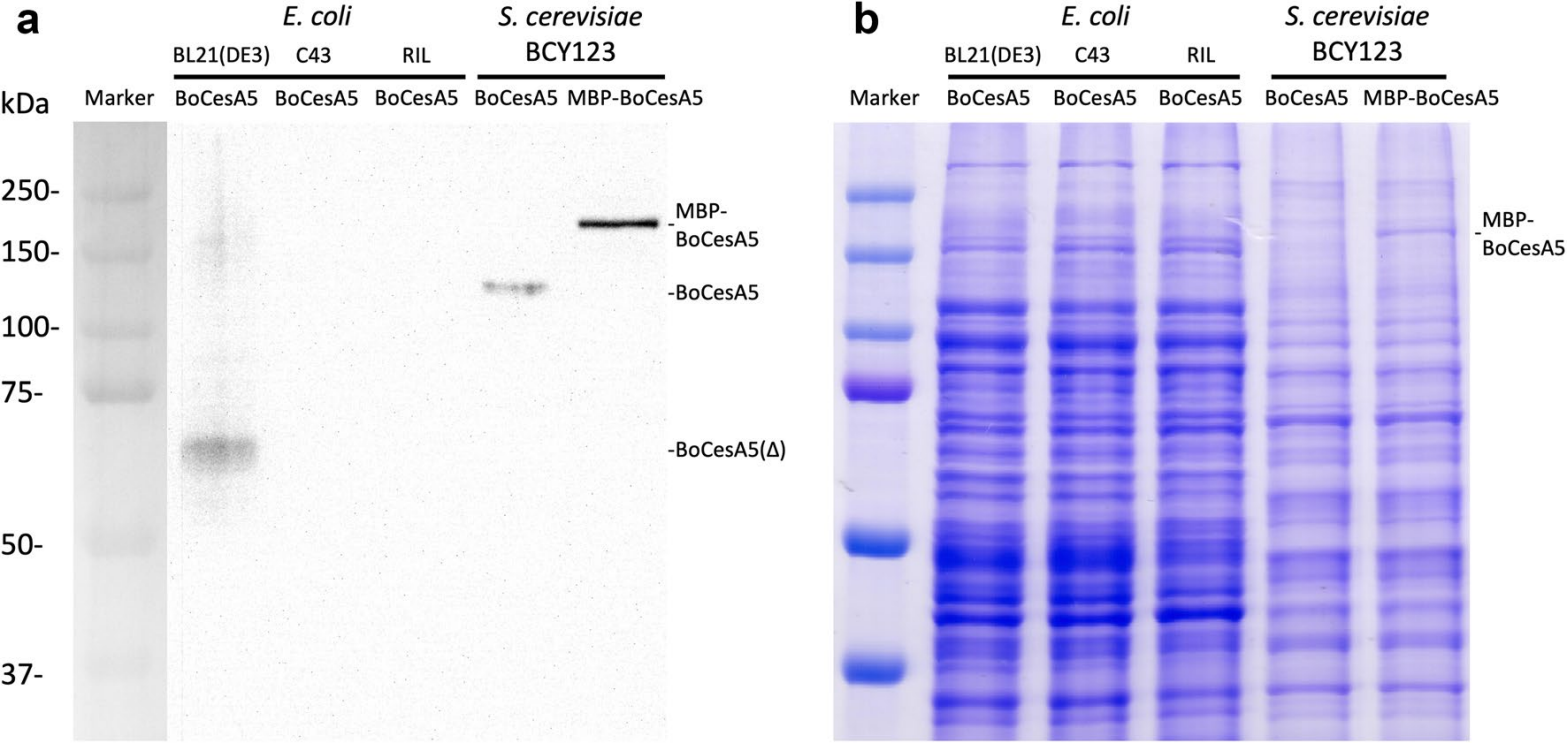
Small-scale expression of BoCesA5 protein. The full-length BoCesA5 and MBP-BoCesA5 were expressed in *S. cerevisiae* while the degraded form BoCesA5(Δ) could be found in *E. coli*. **a** Western blot of BoCesA5 expression in *E. coli* and *S. cerevisiae*, signal detected by anti-BoCesA4/5 polyclonal antibody. **b** Coomassie blue staining of BoCesA5 expression in *E. coli* and *S. cerevisiae*

BoCesA5 is ubiquitously expressed and involved in primary cell wall deposition [28]; thus, we regard BoCesA5 as an indicative gene for expression system screening. We first attempted to express the full-length *BoCesA5* gene in the prokaryotic system, but the *E. coli* strains were not able to express the full-length BoCesA5 protein after examination (Fig. 3). Furthermore, the full-length BoCesA5 protein (120 kDa) under *E. coli* expression resulted in a protein fragment (~ 60 kDa) or invalid expression (Fig. 3a), indicating the BoCesA5 would be unstable in prokaryotic expression.

In the next step, we attempted to use the eukaryotic system for BoCesA5 expression. In western blotting, a stable band of BoCesA5 (~ 120 kDa) was detected by Anti-BoCesA4/5 polyclonal antibody in the total cell lysate (Fig. 3a) after using the yeast strain BCY123 as an expression host. In addition, BoCesA5 conjugated with maltose-binding protein (MBP-BoCesA5) was observed to have a higher expression than full-length BoCesA5. The protein band of MBP-BoCesA5 was easily observed after Coomassie Blue staining (Fig. 3b).

After successful expression of MBP-BoCesA5, we decided to use the same strategy to express the other BoCesAs protein. The constructs of the expression vector were composed of 6×His for column binding, MBP for expression enhancement, T for the TEV protease cleavage site, and CesA genes (*BoCesA1*, *2*, *3*, *4*, *5*, *7*) (Fig. 1). To obtain CesA protein in milligram quantities, we cultured the yeast strain in fed-batch fermentation for 36 h (Fig. 2) to increase the cell density and culture volume.

### BoCesAs conjugated with the MBP tag could be purified at higher amounts

**Fig. 4:**
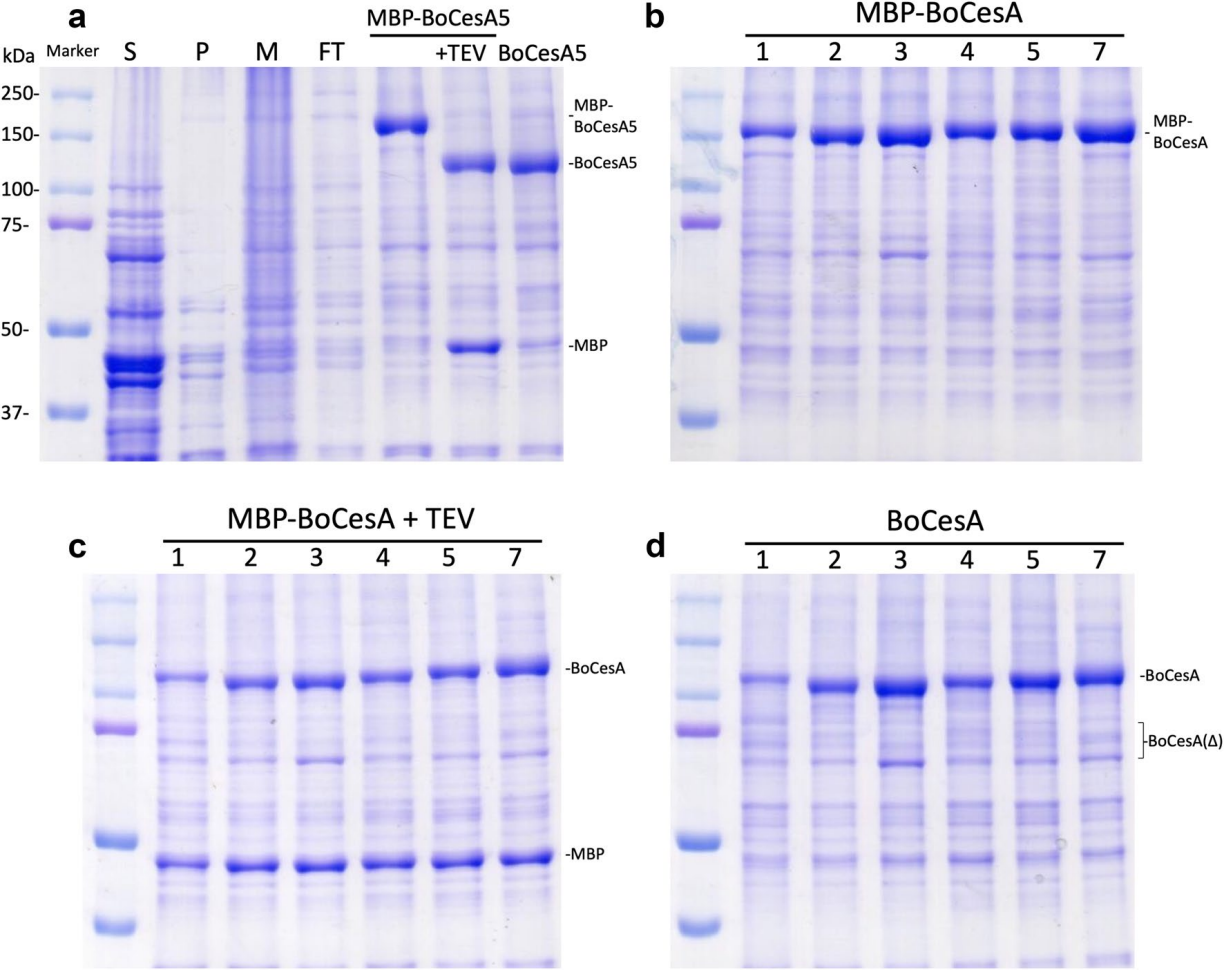
Overview of BoCesA protein purification. **a** Coomassie blue staining during each purification procedure of MBP-BoCesA5. *S* supernatant after ultracentrifugation, *P* pellet of cell debris, *M* membrane solution, *FT* flow through of membrane solution in column binding stage, *MBP-BoCesA5* BoCesA5 with MBP fusion tag (+ TEV: MBP fusion tag cleavage), *BoCesA5* fusion tag removed BoCesA5. **b** Overview of MBP-BoCesA protein purification. The nickel column-purified MBP-BoCesA protein was concentrated to 1.7 mg/mL (10 µM). **c** Overview of MBP-BoCesA protein with TEV protease treatment. MBP-BoCesA could be separated into BoCesA (120 kDa) and MBP (49 kDa) by TEV protease treatment. **d** Overview of BoCesA protein recovery via size exclusion chromatography from Fig. 6. BoCesA recovered from fractions 8–12 was concentrated to ~ 1.2 mg/mL (10 µM). The degradation of BoCesA5(Δ) was identified by western blot in Additional file 3: Figure S3

In brief, the steps involved in MBP-BoCesA protein purification are cell homogenization, solubilization of the MBP-BoCesA protein by mild detergent and purification of the MBP-BoCesA protein by immobilized metal affinity chromatography (IMAC). After cell lysis, partial MBP-BoCesA5 protein was found in the pellet of cell debris of the pretreatment that was discarded. The water-insoluble MBP-BoCesA5 protein was collected in the membrane pellet after ultracentrifugation and was solubilized in dodecyl-maltoside (DDM)-containing buffer for IMAC purification (Fig. 4a, Additional file 1: Figure S1). Pretreatment was performed for other MBP-BoCesA proteins due to the intact MBP-BoCesA5 in the membrane solution.

During the column binding stage of IMAC, massive amounts of yeast proteins remained after column washing with 0.3 M NaCl in the absence of reducing agent (Additional file 2: Figure S2); consequently, we introduced 1 M NaCl and 1 mM TCEP to decrease the amount of contaminants. After elution, MBP-BoCesA proteins with 80% purity were concentrated to ~ 1.7 mg/mL (~ 10 µM) for tag removal (Fig. 4b).

### Cellulose synthase proteins exhibited an oligomeric form after MBP tag removal

After the affinity column purification step, the MBP tags have to be removed from the full-length CesA proteins. Since a TEV protease cutting sequence “ENLYFQ (G/S)” was constructed between MBP and BoCesA, the MBP fusion tag could be removed by adding TEV protease from MBP-BoCesA protein. TEV protease was then added to allow overnight digestion. The major band (MBP-BoCesA) could therefore be separated, and two bands with BoCesA protein and MBP protein were observed using SDS-PAGE (Fig. 4b, c). Further purification by size exclusion chromatography was performed with a FPLC-equipped Superdex-200 column for separating BoCesA, MBP and TEV protease. At the same time, the DDM detergent in the buffer was changed to FC-16 detergent.

**Fig. 5:**
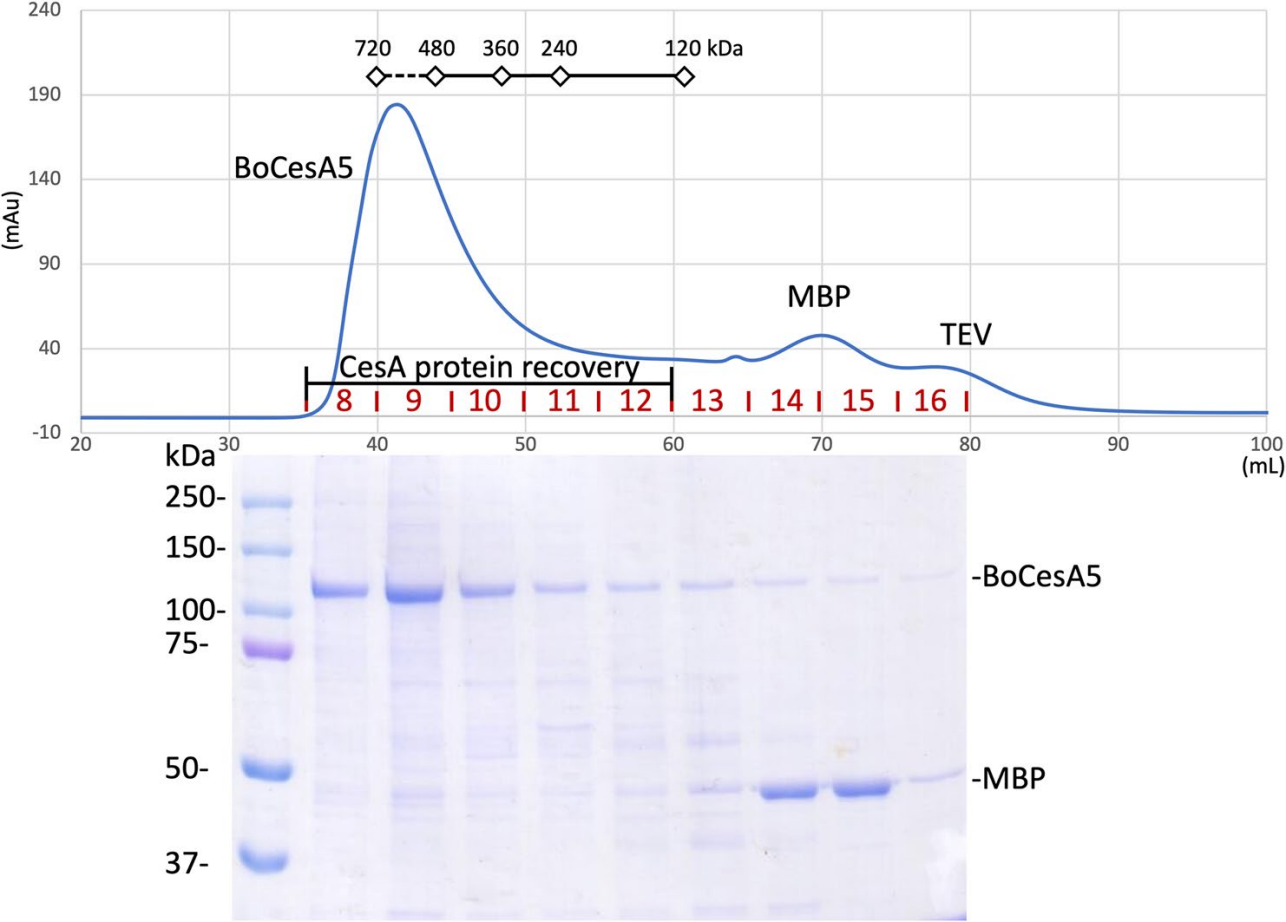
Size-exclusion profile of BoCesA5 protein during secondary purification. After protease treatment, the protein mixture containing BoCesA5, MBP, and TEV protease was injected into a size-exclusion column (Superdex-200) to isolate BoCesA5 according to molecular weight. The SDS-PAGE results corresponded to the size-exclusion profile through fractions 8–16. The main peak, fraction 8–12, was BoCesA5 protein to be recovered. X-axis: elution volume (mL) in size-exclusion column; Y-axis: absorbance (mAu) at 280 nm

**Fig. 6:**
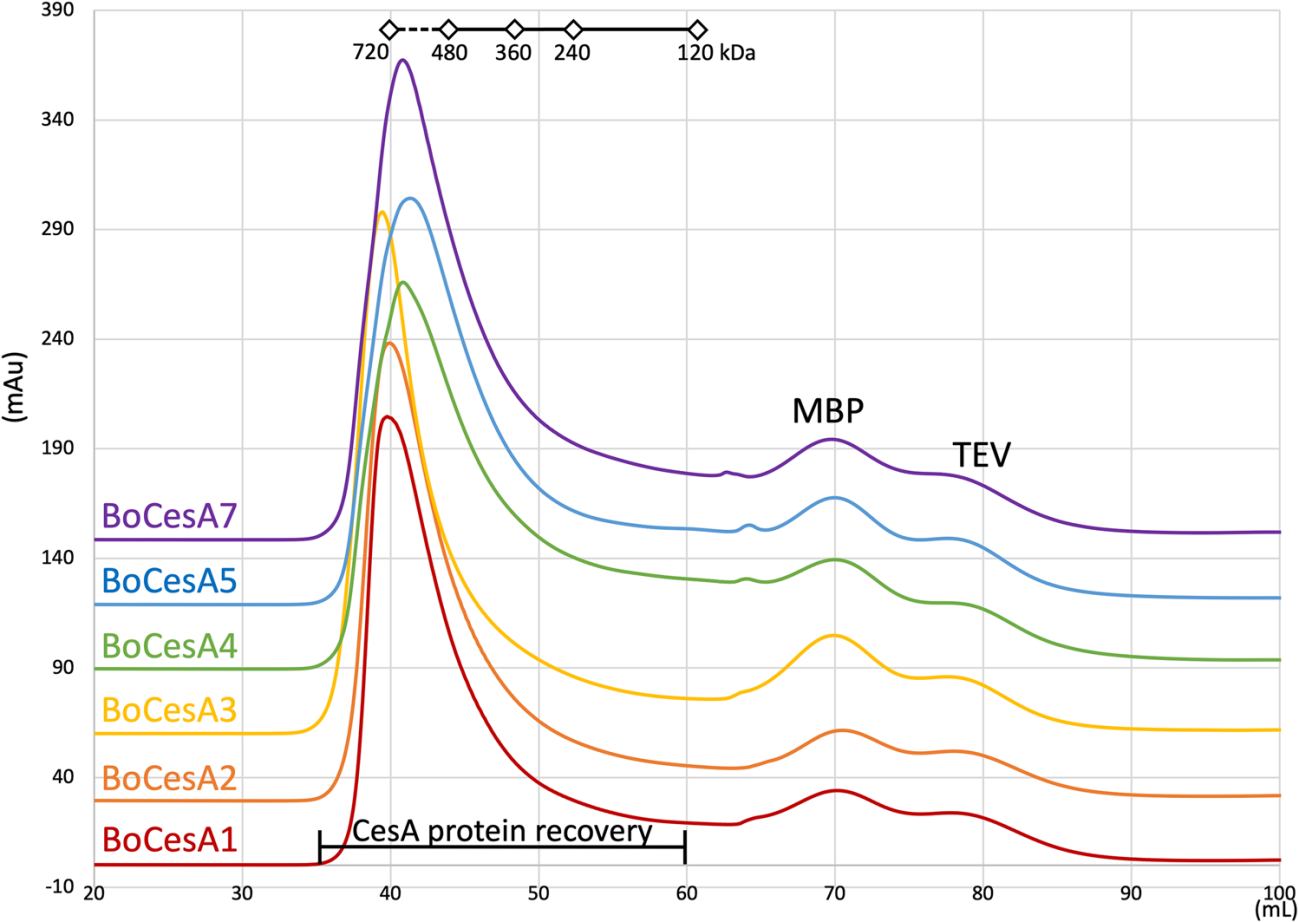
Size-exclusion profile of BoCesA proteins during secondary purification. Overview of BoCesA proteins in size-exclusion profile after protease treatment. The protein mixture containing BoCesA, MBP, and TEV protease was injected into the size-exclusion column to isolate BoCesA according to molecular weight. The main peaks in each profile were BoCesA proteins to be recovered. X-axis: elution volume (mL) in size-exclusion column; Y-axis: absorbance (mAu) at 280 nm

Size exclusion chromatography (SEC) showed that BoCesA5 formed oligomers with a molecular mass that was close to that of the BoCesA5 hexamer (720 kDa) (Fig. 5). These oligomers of BoCesA were also observed by SEC separation after MBP fusion tag removal (Fig. 6). A comparison of the peaks of all purified BoCesAs indicated that the molecular weights of Clade V BoCesA (BoCesA4, 5) and Clade I BoCesA (BoCesA7) were slightly lower than that of Clade VI BoCesA (BoCesA1, 2, 3), indicating that the oligomeric state of BoCesA1, 2, 3, 4, 5, 7 proteins might be a hexamer with slight differences in molecular weight among clades.

**Fig. 7:**
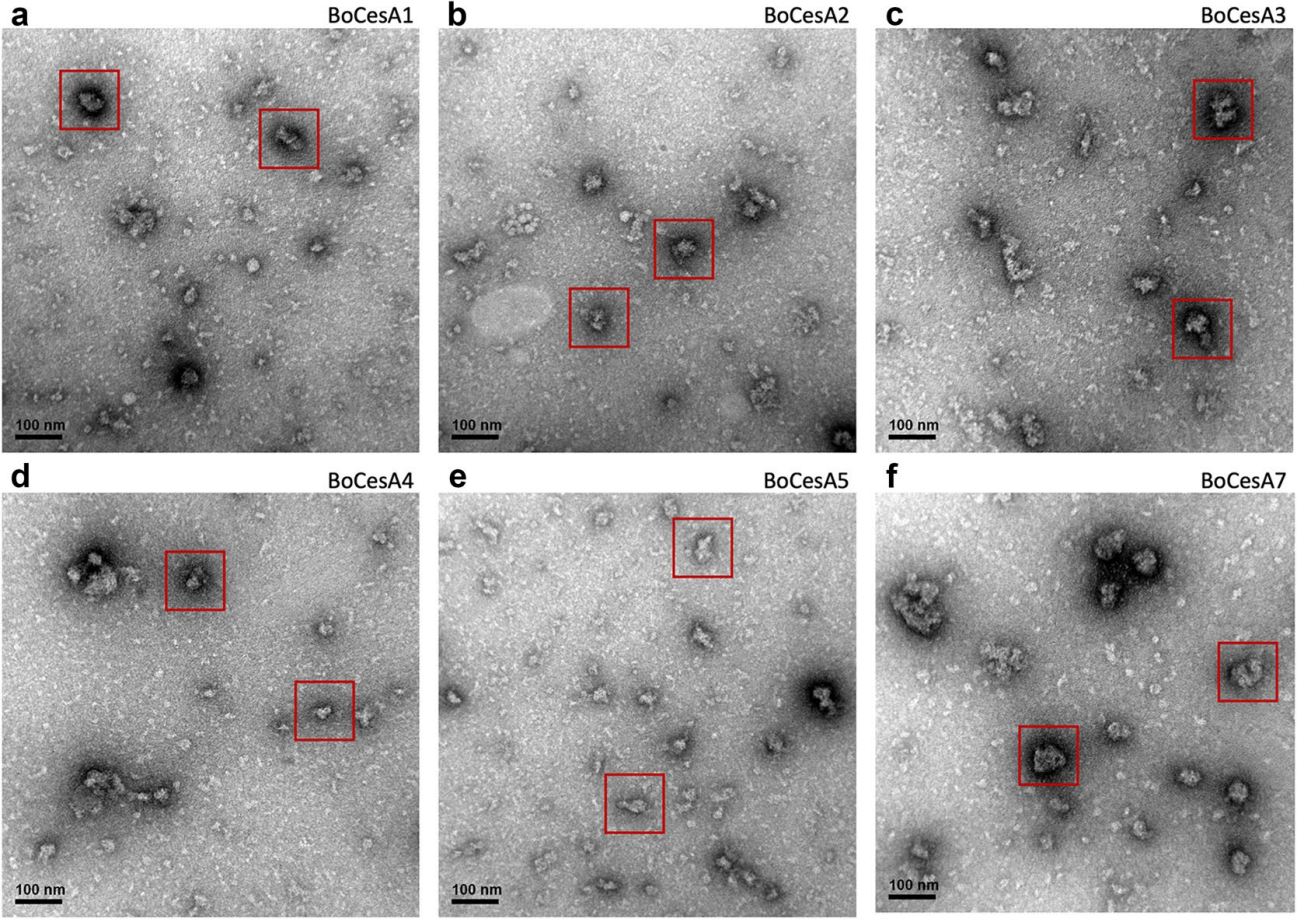
Negative staining images of BoCesA protein particles by TEM. **a**–**c**, **f** Images of the spherically-shaped particles (about 30–50 nm in diameter) obtained from BoCesA1, 2, 3, 7. **d**,** e** The spherical/rod-like shape (about 20–30 nm in diameter) particle images obtained from BoCesA4, 5 (**d**, **e**)

To understand the structure of oligomeric BoCesA proteins, we analyzed their size and shape using TEM images. In the 120-K magnification images, spherically-shaped (approximately 30–50 nm in diameter) particles were observed in Clade VI BoCesA (BoCesA1, 2, 3) and Clade I BoCesA (BoCesA7) (Fig. 7a–c, f) while spherical/rod-shaped (approximately 20–30 nm in diameter) particles were found in Clade V BoCesA (BoCesA4, 5) (Fig. 7d, e).

### Homo-oligomer BoCesAs have catalytic activity

Previous studies showed that recombinant CesA proteins from plants are reconstituted in proteo-liposomes with divalent cations that have enzymatic activity [32, 33]. In this study, we tested the enzyme activity of the recombinant BoCesA proteins that were able to synthesize cellulose in a buffer containing mild detergent. To further confirm the purified BoCesA proteins with enzyme activity, glucan and TEM analyses were used to identify and observe the glucan products.

As endogenous glucan interferes with partially methylated alditol acetate (PMAA)-coupled GC–MS analysis, we first analyzed the endogenous 1,4-linkage glucan in the purified BoCesA proteins. The results showed that the 1,4-linkage glucan signal was present in the purified BoCesA proteins (Additional file 4: Figure S4A–F) and could be removed by amylase digestion (Additional file 4: Figure S4G–L).

**Fig. 8:**
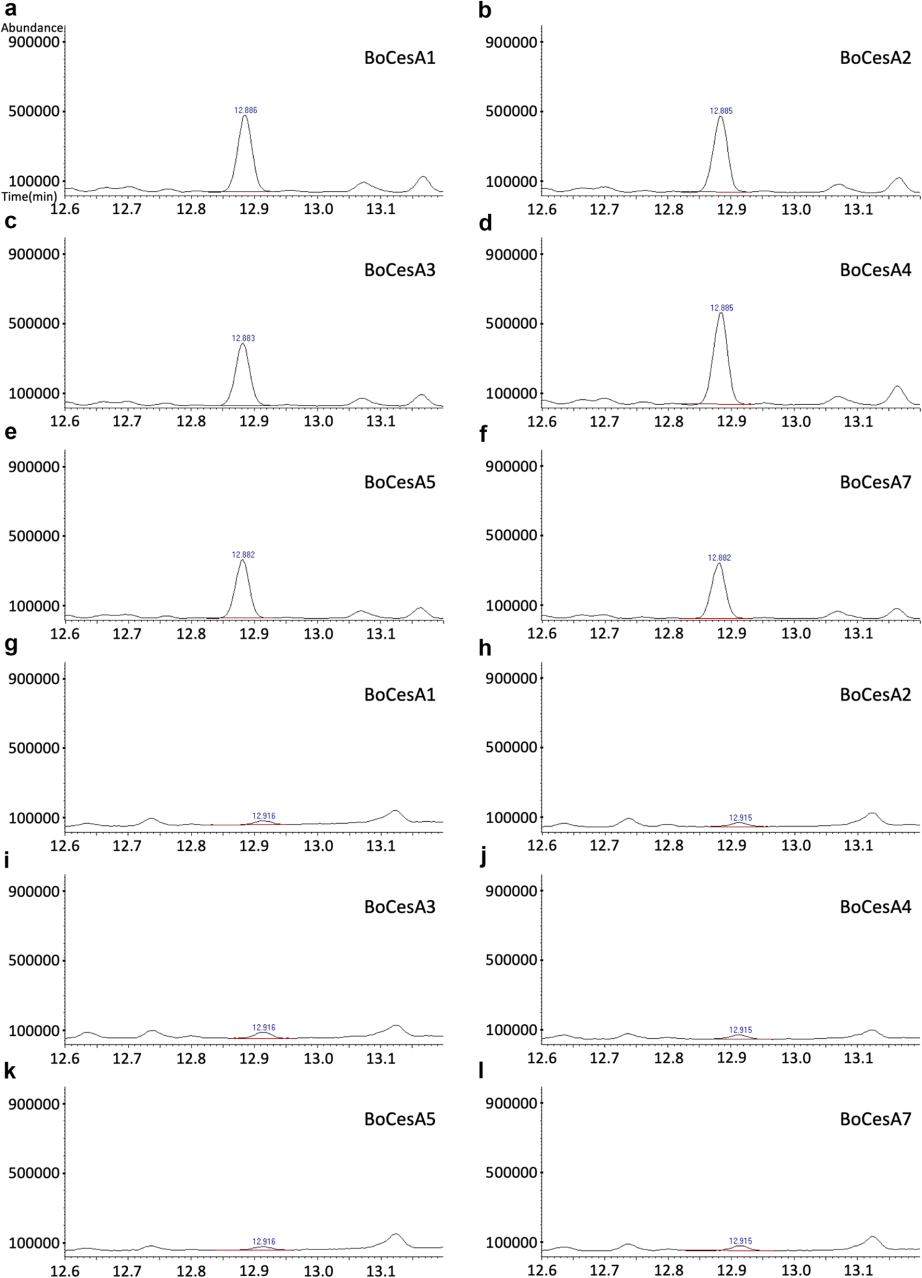
GC-MS total ion chromatogram of 1,4-glucan derivatives from synthesized products. **a**–**f** The peaks of 1,4-glucan derivatives contributed by the product of BoCesA1, 2, 3, 4, 5, 7. The mass spectra are shown in (Additional file 5: Figure S5). **g**–**l** The peaks of 1,4-glucan derivatives contributed by the product of BoCesA1, 2, 3, 4, 5, 7 after cellulase treatment

Subsequently, in vitro synthesized 1,4-glucan chains were determined by oligomer BoCesA incubated with UDP-Glc and divalent manganese-coupled amylase treatment. The synthesized 1,4-glucan in each reaction (BoCesA1, 2, 3, 4, 5, 7) could be detected after amylase treatment (Fig. 8a–f) from which the endogenous α-1,4-glucan had been removed. The signal from the synthesized 1,4-glucan was reduced after the cellulase treatment (Fig. 8g–l), indicating that the in vitro* s*ynthesized product should be a β-1,4-glucan. The results are consistent with those from previous studies [32, 33] in indicating that the single isoform of plant cellulose synthase can produce β-1,4-glucan chains.

**Fig. 9:**
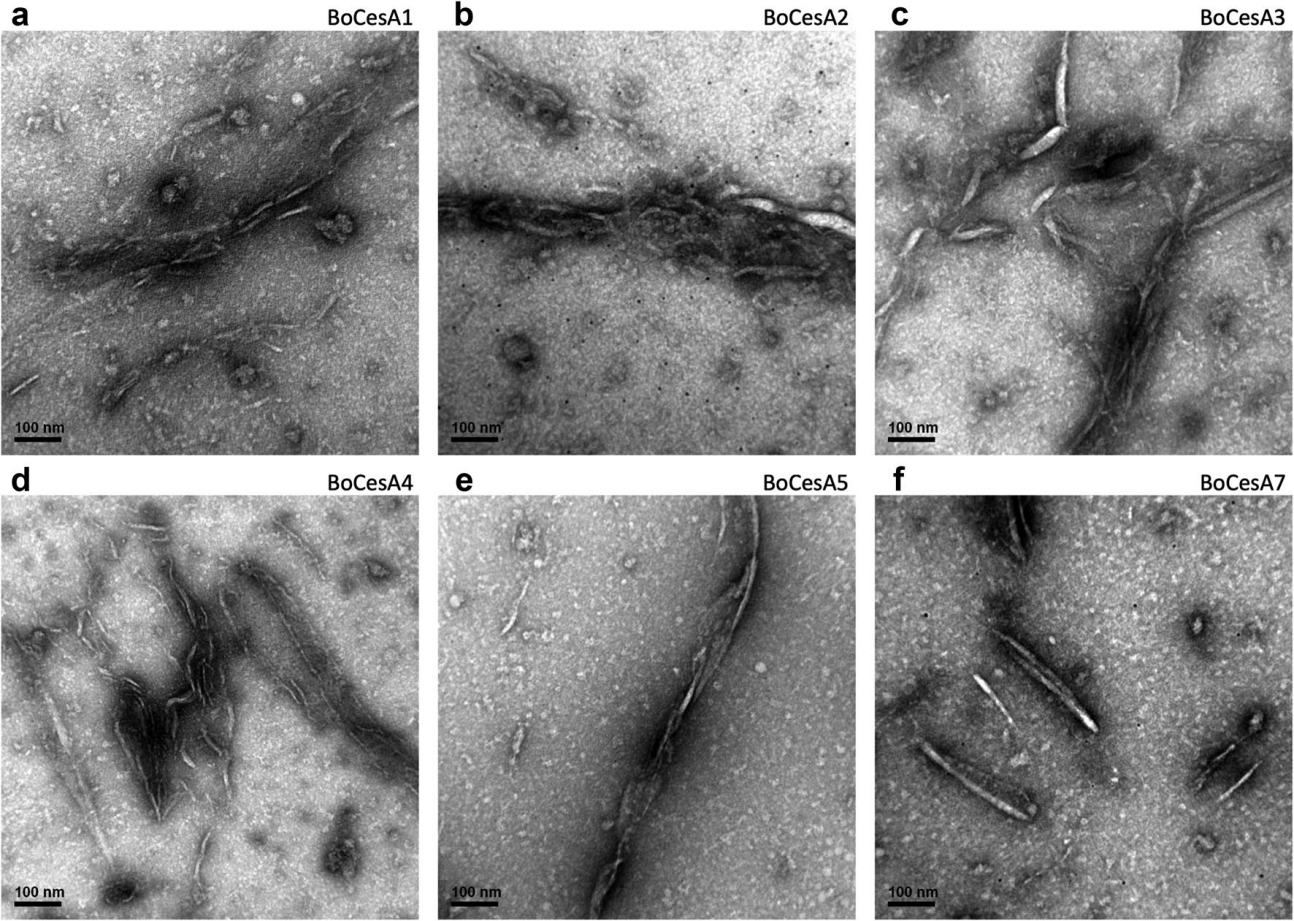
Negative staining images of BoCesA synthesized product by TEM. **a**–**f** The synthesized product from BoCesA1, 2, 3, 4, 5, 7

Lastly, the synthesized product from the in vitro assay was observed using TEM. Within a 3 h incubation, a substance with a linear/twisted form appeared and formed a microfibril structure under negative staining (Fig. 9a–f). This substance decreased or disappeared with cellulase treatment (Additional file 6: Figure S6A–F); consequently, we concluded that the substance was likely cellulose microfibrils.

## Discussion

In this study, the cellulose synthases from green bamboo were heterologously expressed and purified. High yields (10 µM) of purified BoCesA proteins with catalytic activity were obtained. Their expression level could be increased by conjugating the MBP tag, and the yield of yeast cells by using a fed-batch fermenter to generate 120 g of yeast cells with a recovery of 7.2 mg BoCesA.

In the *E. coli* strain BL21(DE3), ~ 60 kDa protein fragments were detected instead of the full-length 120 kDa CesA protein, suggesting that the CesA protein expressed in the BL21(DE3) strain can easily be degraded. We determined that the full-length CesA protein also cannot be expressed in two other *E. coli* strains (C43 and RIL) that featuring in the membrane protein expression and the codon usage bias, respectively. In previous studies, the CesA protein was delivered to the plasma membrane by the Golgi apparatus within vesicles called "small CESA compartments (SmaCCs) or microtubule-associated cellulose synthase compartments (MASCs)" [34, 35]. It is understood that *E. coli* lacks several compartments required for the delivery of the CesA protein. Others have also shown that its successful expression in prokaryotes results in the truncated form of CesA [36, 37] instead of the full-length CesA. However, the full-length CesA protein was successfully expressed in yeast, which we utilized to produce 6 BoCesA proteins in this study. Furthermore, fusing target proteins to a maltose-binding protein has been shown to increase the expression and folding of eukaryotic fusion proteins [38].

Previous studies demonstrated that the cell membrane can be destroyed by adding harsh detergent to terminate cellulose synthesis in vitro [32, 33]. However, the multiple transmembrane helices-tunneled polysaccharide synthase may allow enzyme activity in a membrane-bound state or detergent solution state. For example, hyaluronan synthase is active in membrane reconstitution and detergent solubilization states [39]. Here, we showed that BoCesA cellulose synthases remain active in the presence of mild detergent.

In glucan analysis, α/β-1,4-glucan will result in an identical derivative compound in the PMAA preparation. Therefore, endogenous α-1,4-glucan will interfere with glucan analysis. The presence of endogenous α-1,4-glucan in BoCesA protein samples might derive from the maltoside of DDM detergent (Additional file 7: Figure S7) remaining after SEC purification. To determine the presence of synthesized β-1,4-glucan using PMAA-coupled GC–MS, amylase was added to remove α-1,4-glucan as in a previous study [32]. The final products could be confirmed as β-1,4-glucans.

Correlation of the terminal complex (hexagonal rosettes) with cellulose microfibril synthesis was established some time ago using freeze-fracture images [14, 15, 40]. These membrane complexes were subsequently discovered to be composed of neatly arranged cellulose synthases [15], which are believed to individually synthesize single glucan chains in the CSC to generate a bundled structure of cellulose microfibrils [41]. In the 36-unit CSC model proposed, the 36-cellulose chain elementary fibril was identified in the cross section of the cellulose microfibril in maize [16]. On the other hand, the image modeling-based 18-unit CSC model in *P. patens* revealed a homo-trimer complex [42], indicating the existence of numerous possibilities for the exact number and arrangement of CesA proteins in CSC.

The CSC diameter has been identified during in vitro cellulose synthesis in *P. patens* membranes [43]; globular particles measuring 40–50 nm were found to be associated with synthesized cellulose microfibrils. In addition, heterologously expressed PttCesA8 with an estimated diameter of 25 nm was observed during in vitro cellulose synthesis [33]. Based on size-exclusion chromatography, the molecular weight of BoCesA proteins was similar to that of a hexamer, with slight differences among clades. Based on TEM observation, the particle sizes and shapes of BoCesA proteins were also distinct; both Clade VI BoCesA (BoCesA1, 2, 3) and Clade I BoCesA (BoCesA7) had a spherical shape measuring 30–50 nm, whereas Clade V BoCesA (BoCesA4, 5) had a spherical/rod-like shape measuring 20–30 nm, implying variations in the quaternary structure of the BoCesA hexamer.

## Conclusions

In summary, we have developed a heterologous expression and purification protocol for BoCesAs. The milligram quantities membrane protein of BoCesAs can be further used for functional and structural studies. The protein yield could be increased by conjugating N-terminal MBP tag, expressed in yeast and cultured with the fed-batch fermenter. The two-step purification procedure, consisting of immobilized metal affinity chromatography and size-exclusion chromatography were employed for BoCesA recombinant proteins purification to isolate BoCesAs in oligomeric form. After the MBP fusion tag removal, the molecular weights of BoCesAs were close to hexamer, and the molecular shape of BoCesA1, 2, 3, 7 and BoCesA4, 5 showed spherical- and spherical/rod-structure, respectively. The catalytic activity of BoCesAs could be detected in a buffer with the detergent FC-16 and the product β-1,4-glucan was observed as microfibril structure.

## Additional files

**Additional file 1: Figure S1.** Immunoblot results of BoCesA5 purification.

**Additional file 2: Figure S2.** Protein purification results in low salt/non-reducing agent condition.

**Additional file 3: Figure S3.** Immunoblot results of purification.

**Additional file 4: Figure S4.** GC–MS total ion chromatogram of 1,4-glucan derivatives form endogenous glucan.

**Additional file 5: Figure S5.** GC–MS mass spectrum of 1,4-glucan derivatives peak in Additional file 4: Figure S4 and Fig. 8.

**Additional file 6: Figure S6.** Negative staining of BoCesA synthesized product after cellulase treatment in TEM.

**Additional file 7: Figure S7.** GC–MS total ion chromatogram and mass spectrum of 1,4-glucan derivatives form *n*-Dodecyl-β-d-maltopyranoside (DDM).

## Data Availability

The experimental materials during the current study are available from the corresponding author on reasonable request.

## Abbreviations

CSC: cellulose synthase complex
CesA: cellulose synthase
MBP: maltose-binding protein
TEV: tobacco etch virus
TEM: transmission electron microscopy
PMAA: partially methylated alditol acetates
GC–MS: gas chromatography–mass spectrometry

## References

[1] Ververis C, Georghiou K, Christodoulakis N, Santas P, Santas R (2004). Fiber dimensions, lignin and cellulose content of various plant materials and their suitability for paper production. Ind Crops Prod.

[2] Sannigrahi P, Ragauskas AJ, Tuskan GA (2010). Poplar as a feedstock for biofuels: a review of compositional characteristics. Biofuels Bioprod Biorefining.

[3] Hong C, Fang J, Jin A, Cai J, Guo H, Ren J, Shao Q, Zheng B (2011). Comparative growth, biomass production and fuel properties among different perennial plants, bamboo and miscanthus. Bot Rev.

[4] Li Z, Jiang Z, Fei B, Cai Z, Pan X (2014). Comparison of bamboo green, timber and yellow in sulfite, sulfuric acid and sodium hydroxide pretreatments for enzymatic saccharification. Biores Technol.

[5] Peng Zhenhua, Lu Ying, Li Lubin, Zhao Qiang, Feng Qi, Gao Zhimin, Lu Hengyun, Hu Tao, Yao Na, Liu Kunyan, Li Yan, Fan Danlin, Guo Yunli, Li Wenjun, Lu Yiqi, Weng Qijun, Zhou CongCong, Zhang Lei, Huang Tao, Zhao Yan, Zhu Chuanrang, Liu Xinge, Yang Xuewen, Wang Tao, Miao Kun, Zhuang Caiyun, Cao Xiaolu, Tang Wenli, Liu Guanshui, Liu Yingli, Chen Jie, Liu Zhenjing, Yuan Licai, Liu Zhenhua, Huang Xuehui, Lu Tingting, Fei Benhua, Ning Zemin, Han Bin, Jiang Zehui (2013). The draft genome of the fast-growing non-timber forest species moso bamboo (Phyllostachys heterocycla). Nature Genetics.

[6] Peng Z, Zhang C, Zhang Y, Hu T, Mu S, Li X, Gao J (2013). Transcriptome sequencing and analysis of the fast growing shoots of moso bamboo (*Phyllostachys edulis*). PLoS ONE.

[7] He CY, Cui K, Zhang JG, Duan AG, Zeng YF (2013). Next-generation sequencing-based mRNA and microRNA expression profiling analysis revealed pathways involved in the rapid growth of developing culms in Moso bamboo. BMC Plant Biol.

[8] Pauly M, Keegstra K (2010). Plant cell wall polymers as precursors for biofuels. Curr Opin Plant Biol.

[9] Nishiyama Y (2009). Structure and properties of the cellulose microfibril. J Wood Sci.

[10] Paredez AR, Somerville CR, Ehrhardt DW (2006). Visualization of cellulose synthase demonstrates functional association with microtubules. Science.

[11] Lairson LL, Henrissat B, Davies GJ, Withers SG (2008). Glycosyltransferases: structures, functions, and mechanisms. Annu Rev Biochem.

[12] Delmer DP (1999). Cellulose biosynthesis: exciting times for a difficult field of study. Annu Rev Plant Phys.

[13] Doblin MS, Kurek I, Jacob-Wilk D, Delmer DP (2002). Cellulose biosynthesis in plants: from genes to rosettes. Plant Cell Physiol.

[14] Mueller SC, Brown RM (1980). Evidence for an intramembrane component associated with a cellulose microfibril-synthesizing complex in higher plants. J Cell Biol.

[15] Herth W (1983). Arrays of plasma-membrane "rosettes" involved in cellulose microfibril formation of *Spirogyra*. Planta.

[16] Ding SY, Himmel ME (2006). The maize primary cell wall microfibril: a new model derived from direct visualization. J Agric Food Chem.

[17] Sethaphong L, Haigler CH, Kubicki JD, Zimmer J, Bonetta D, DeBolt S, Yingling YG (2013). Tertiary model of a plant cellulose synthase. Proc Natl Acad Sci.

[18] Morgan JL, Strumillo J, Zimmer J (2013). Crystallographic snapshot of cellulose synthesis and membrane translocation. Nature.

[19] Pear JR, Kawagoe Y, Schreckengost WE, Delmer DP, Stalker DM (1996). Higher plants contain homologs of the bacterial celA genes encoding the catalytic subunit of cellulose synthase. Proc Natl Acad Sci USA.

[20] Somerville C (2006). Cellulose synthesis in higher plants. Annu Rev Cell Dev Biol.

[21] Rushton PS, Olek AT, Makowski L, Badger J, Steussy CN, Carpita NC, Stauffacher CV (2017). Rice Cellulose SynthaseA8 plant-conserved region is a coiled-coil at the catalytic core entrance. Plant Physiol.

[22] Richmond T (2000). Higher plant cellulose synthases. Genome Biol..

[23] Taylor NG, Laurie S, Turner SR (2000). Multiple cellulose synthase catalytic subunits are required for cellulose synthesis in *Arabidopsis*. Plant Cell.

[24] Scheible WR, Eshed R, Richmond T, Delmer D, Somerville C (2001). Modifications of cellulose synthase confer resistance to isoxaben and thiazolidinone herbicides in *Arabidopsis* Ixr1 mutants. Proc Natl Acad Sci USA.

[25] Desprez T, Juraniec M, Crowell EF, Jouy H, Pochylova Z, Parcy F, Hofte H, Gonneau M, Vernhettes S (2007). Organization of cellulose synthase complexes involved in primary cell wall synthesis in *Arabidopsis thaliana*. Proc Natl Acad Sci USA.

[26] Timmers J, Vernhettes S, Desprez T, Vincken JP, Visser RG, Trindade LM (2009). Interactions between membrane-bound cellulose synthases involved in the synthesis of the secondary cell wall. FEBS Lett.

[27] Taylor NG, Howells RM, Huttly AK, Vickers K, Turner SR (2003). Interactions among three distinct CesA proteins essential for cellulose synthesis. Proc Natl Acad Sci USA.

[28] Chen CY, Hsieh MH, Yang CC, Lin CS, Wang AY (2010). Analysis of the cellulose synthase genes associated with primary cell wall synthesis in *Bambusa oldhamii*. Phytochemistry.

[29] Kapust RB, Waugh DS (1999). *Escherichia coli* maltose-binding protein is uncommonly effective at promoting the solubility of polypeptides to which it is fused. Protein Sci.

[30] Chen C-Y. Analysis of the cellulose synthase genes associated with primary cell wall synthesis in *Bambusa oldhamii*. Doctoral Dissertation*.* Taipei, Taiwan: National Taiwan University; 2010. 10.1016/j.phytochem.2010.05.011 20541781

[31] Pettolino FA, Walsh C, Fincher GB, Bacic A (2012). Determining the polysaccharide composition of plant cell walls. Nat Protoc.

[32] Cho SH, Purushotham P, Fang C, Maranas C, Diaz-Moreno SM, Bulone V, Zimmer J, Kumar M, Nixon BT (2017). Synthesis and self-assembly of cellulose microfibrils from reconstituted cellulose synthase. Plant Physiol.

[33] Purushotham P, Cho SH, Díaz-Moreno SM, Kumar M, Nixon BT, Bulone V, Zimmer J (2016). A single heterologously expressed plant cellulose synthase isoform is sufficient for cellulose microfibril formation in vitro. Proc Natl Acad Sci..

[34] Wightman R, Turner S (2010). Trafficking of the plant cellulose synthase complex. Plant Physiol.

[35] Crowell EF, Gonneau M, Stierhof YD, Hofte H, Vernhettes S (2010). Regulated trafficking of cellulose synthases. Curr Opin Plant Biol.

[36] Vandavasi VG, Putnam DK, Zhang Q, Petridis L, Heller WT, Nixon BT, Haigler CH, Kalluri U, Coates L, Langan P (2016). A structural study of CESA1 catalytic domain of *Arabidopsis* cellulose synthesis complex: evidence for CESA trimers. Plant Physiol.

[37] Olek AT, Rayon C, Makowski L, Kim HR, Ciesielski P, Badger J, Paul LN, Ghosh S, Kihara D, Crowley M (2014). The structure of the catalytic domain of a plant cellulose synthase and its assembly into dimers. Plant Cell.

[38] Young CL, Britton ZT, Robinson AS (2012). Recombinant protein expression and purification: a comprehensive review of affinity tags and microbial applications. Biotechnol J.

[39] Blackburn MR, Hubbard C, Kiessling V, Bi Y, Kloss B, Tamm LK, Zimmer J (2018). Distinct reaction mechanisms for hyaluronan biosynthesis in different kingdoms of life. Glycobiology.

[40] Reiss HD, Schnepf E, Herth W (1984). The plasma membrane of the *Funaria* caulonema tip cell: morphology and distribution of particle rosettes, and the kinetics of cellulose synthesis. Planta.

[41] Newman RH, Hill SJ, Harris PJ (2013). Wide-angle x-ray scattering and solid-state nuclear magnetic resonance data combined to test models for cellulose microfibrils in mung bean cell walls. Plant Physiol.

[42] Nixon BT, Mansouri K, Singh A, Du J, Davis JK, Lee JG, Slabaugh E, Vandavasi VG, O'Neill H, Roberts EM (2016). Comparative structural and computational analysis supports eighteen cellulose synthases in the plant cellulose synthesis complex. Sci Rep.

[43] Cho SH, Du J, Sines I, Poosarla VG, Vepachedu V, Kafle K, Park YB, Kim SH, Kumar M, Nixon BT (2015). In vitro synthesis of cellulose microfibrils by a membrane protein from protoplasts of the non-vascular plant Physcomitrella patens. Biochem J.

