# Supplementary material for: Heterologous overexpression, purification and functional analysis of plant cellulose synthase from green bamboo
Source: Plant Methods. 2019 Jul 25;15:80. doi: 10.1186/s13007-019-0466-0 (PMC6657065; doi:10.1186/s13007-019-0466-0)
Supplement: Supplementary file 1 — Additional file 1: Figure S1. Immunoblot results of BoCesA5 purification. [file 13007_2019_466_MOESM1_ESM.pdf]

Figure S1

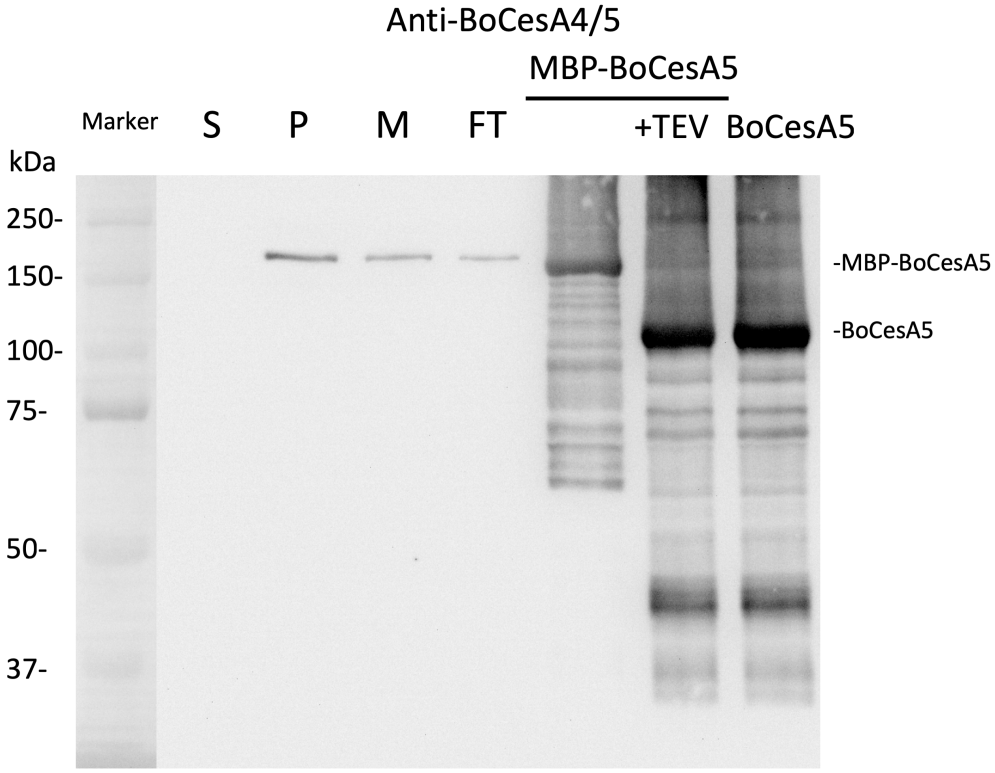

Figure S1. Immunoblot results of BoCesA5 purification.

The immunoblot during each purification procedure of MBP-BoCesA5 in Fig. 4a, signal detected by Anti-BoCesA4/5 polyclonal antibody.
