# Supplementary material for: Heterologous overexpression, purification and functional analysis of plant cellulose synthase from green bamboo
Source: Plant Methods. 2019 Jul 25;15:80. doi: 10.1186/s13007-019-0466-0 (PMC6657065; doi:10.1186/s13007-019-0466-0)
Supplement: Supplementary file 2 — Additional file 2: Figure S2. Protein purification results in low salt/non-reducing agent condition. [file 13007_2019_466_MOESM2_ESM.pdf]

Figure S2

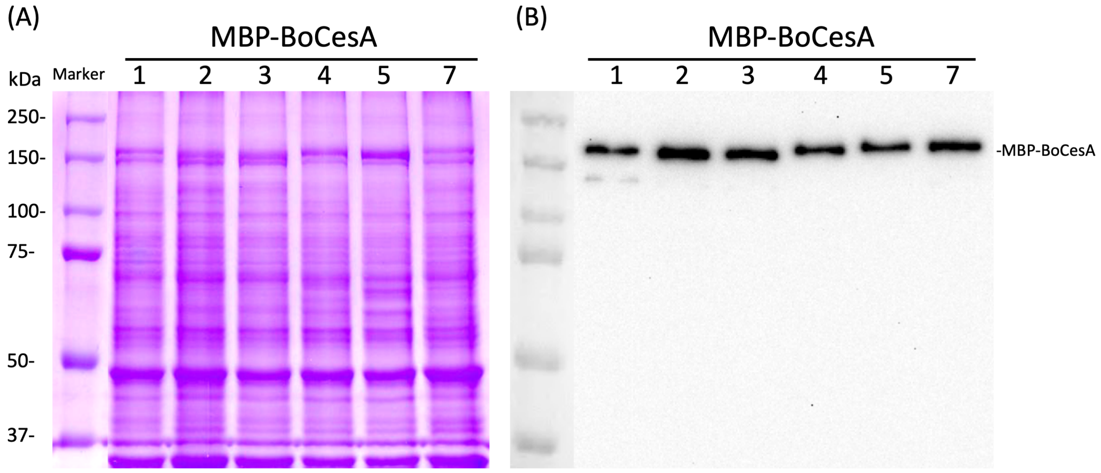

Figure S2. Protein purification results in low salt/non-reducing agent condition.

The MBP-BoCesA would be purified with many contaminates. **A**: Coomassive blue staining of BoCesA protein elution. **B**: Western blot of BoCesA protein elution, signal detected by MBP antibody.
