# Supplementary material for: Heterologous overexpression, purification and functional analysis of plant cellulose synthase from green bamboo
Source: Plant Methods. 2019 Jul 25;15:80. doi: 10.1186/s13007-019-0466-0 (PMC6657065; doi:10.1186/s13007-019-0466-0)
Supplement: Supplementary file 3 — Additional file 3: Figure S3. Immunoblot results of purification. [file 13007_2019_466_MOESM3_ESM.pdf]

Figure S3

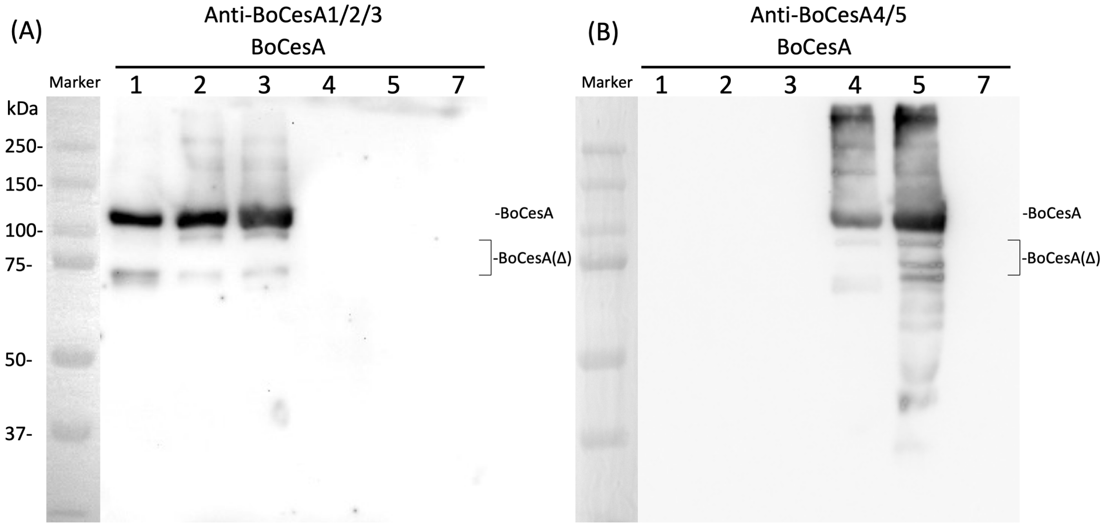

Figure S3. Immunoblot results of purification.

A: The immunoblot of BoCesA in Fig. 4d, signal detected by Anti-BoCesA1/2/3 polyclonal antibody. **B**: The immunoblot of BoCesA in Fig. 4d, signal detected by Anti-BoCesA4/5 polyclonal antibody.
