# Supplementary material for: Heterologous overexpression, purification and functional analysis of plant cellulose synthase from green bamboo
Source: Plant Methods. 2019 Jul 25;15:80. doi: 10.1186/s13007-019-0466-0 (PMC6657065; doi:10.1186/s13007-019-0466-0)
Supplement: Supplementary file 4 — Additional file 4: Figure S4. GC–MS total ion chromatogram of 1,4-glucan derivatives form endogenous glucan. [file 13007_2019_466_MOESM4_ESM.pdf]

Figure S4

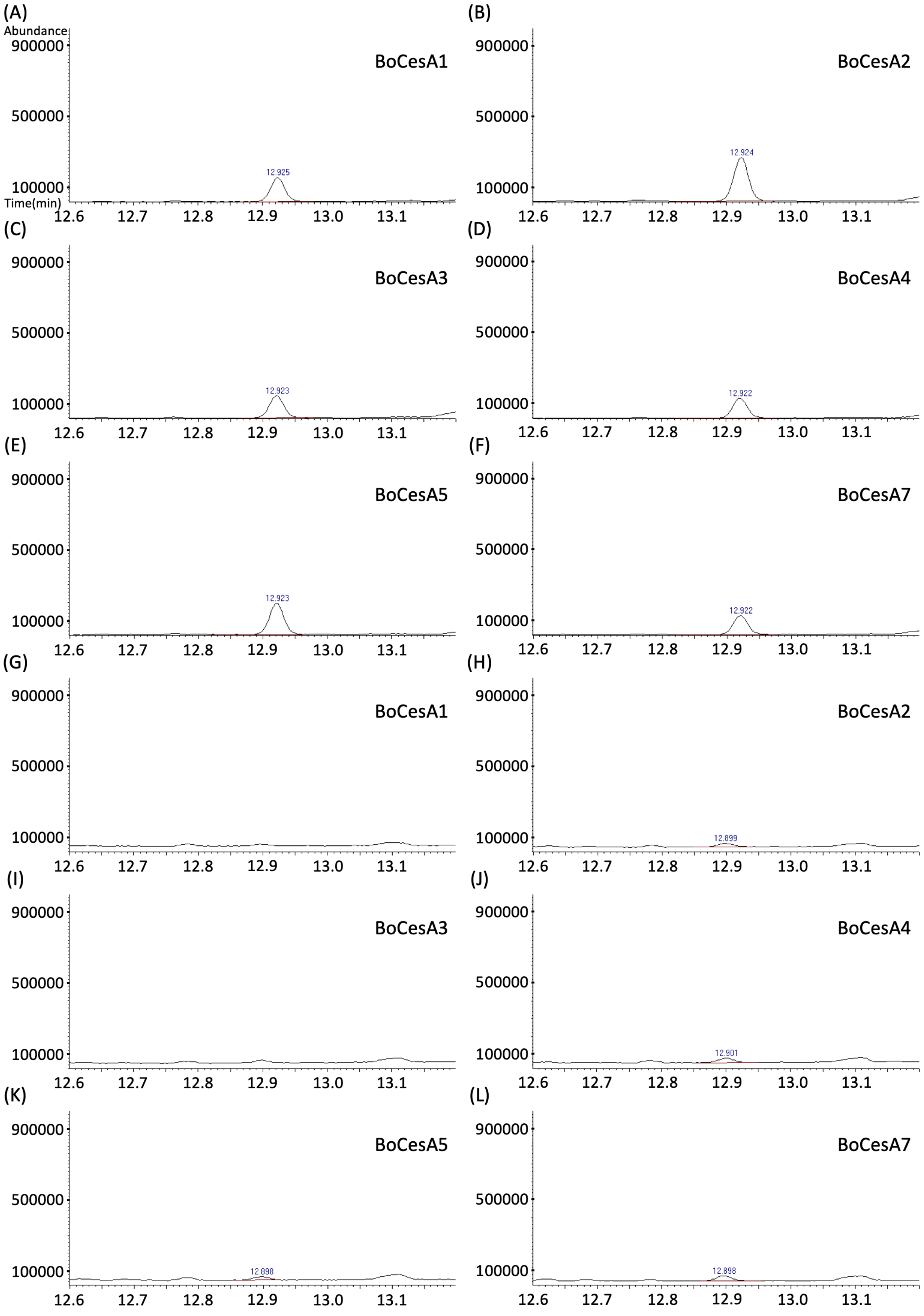

Figure S4. GC-MS total ion chromatogram of 1,4-glucan derivatives form endogenous glucan.

A-F: The peaks of 1,4-glucan derivatives contributed by endogenous glucan of BoCesA1, 2, 3, 4, 5, 7. The mass spectra are shown in (Additional file 5: Figure S5). **G-L**: The peaks of 1,4-glucan derivatives contributed by endogenous glucan of BoCesA1, 2, 3, 4, 5, 7 with amylase treatment.
