# Supplementary material for: Heterologous overexpression, purification and functional analysis of plant cellulose synthase from green bamboo
Source: Plant Methods. 2019 Jul 25;15:80. doi: 10.1186/s13007-019-0466-0 (PMC6657065; doi:10.1186/s13007-019-0466-0)
Supplement: Supplementary file 5 — Additional file 5: Figure S5. GC–MS mass spectrum of 1,4-glucan derivatives peak in Additional file 4: Figure S4 and Fig. 8. [file 13007_2019_466_MOESM5_ESM.pdf]

Figure S5

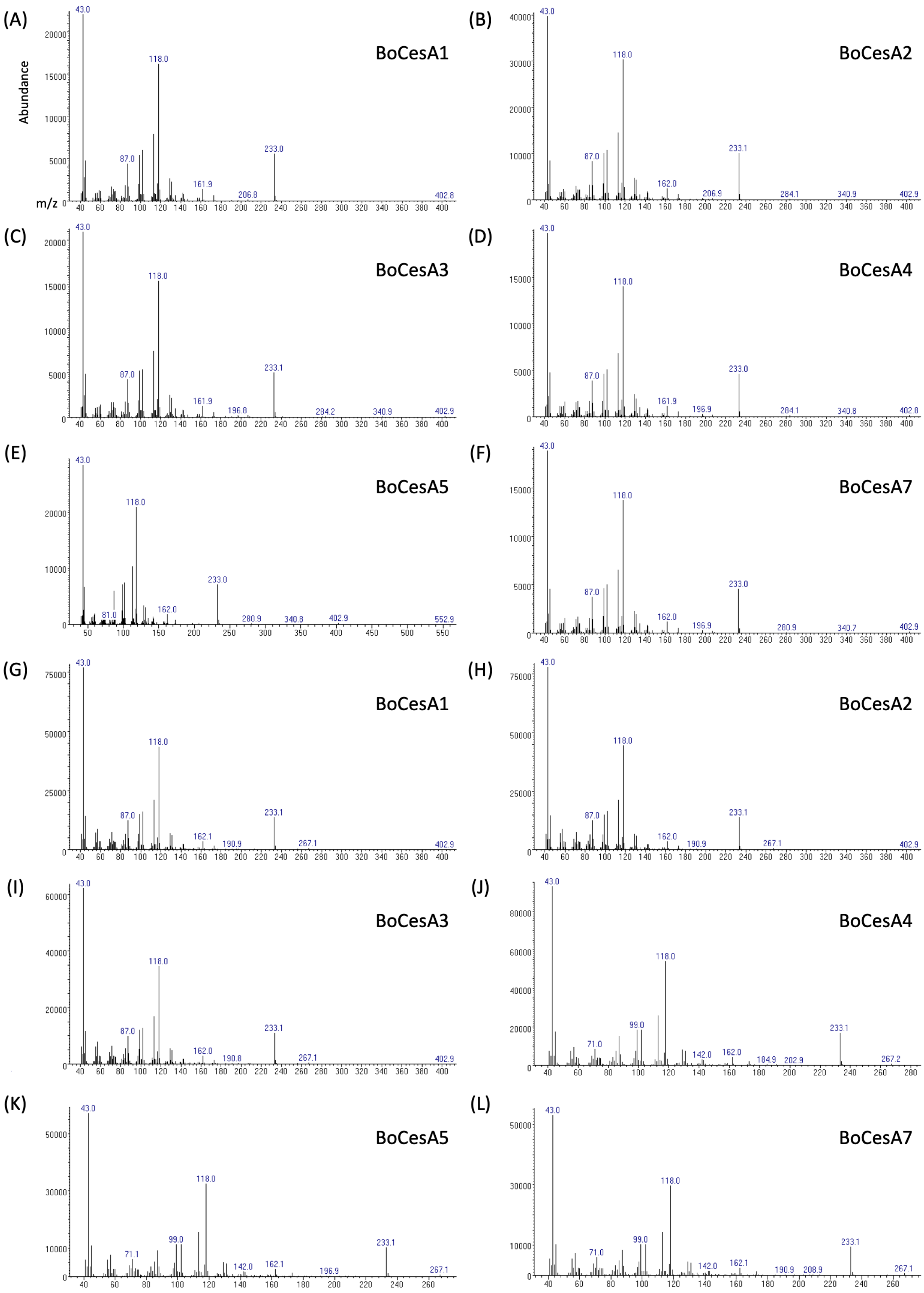

Figure S5. GC-MS mass spectrum of 1,4-glucan derivatives peak in Additional file 4: Figure S4 and Fig. 8.

A-F: The mass spectrum of 1,4-glucan derivatives peak in Additional file 4: Figure S4A-F. **G-L**: The mass spectrum of 1,4-glucan derivatives peak in Fig. 8a-f.
