# Supplementary material for: Heterologous overexpression, purification and functional analysis of plant cellulose synthase from green bamboo
Source: Plant Methods. 2019 Jul 25;15:80. doi: 10.1186/s13007-019-0466-0 (PMC6657065; doi:10.1186/s13007-019-0466-0)
Supplement: Supplementary file 6 — Additional file 6: Figure S6. Negative staining of BoCesA synthesized product after cellulase treatment in TEM. [file 13007_2019_466_MOESM6_ESM.pdf]

Figure S6

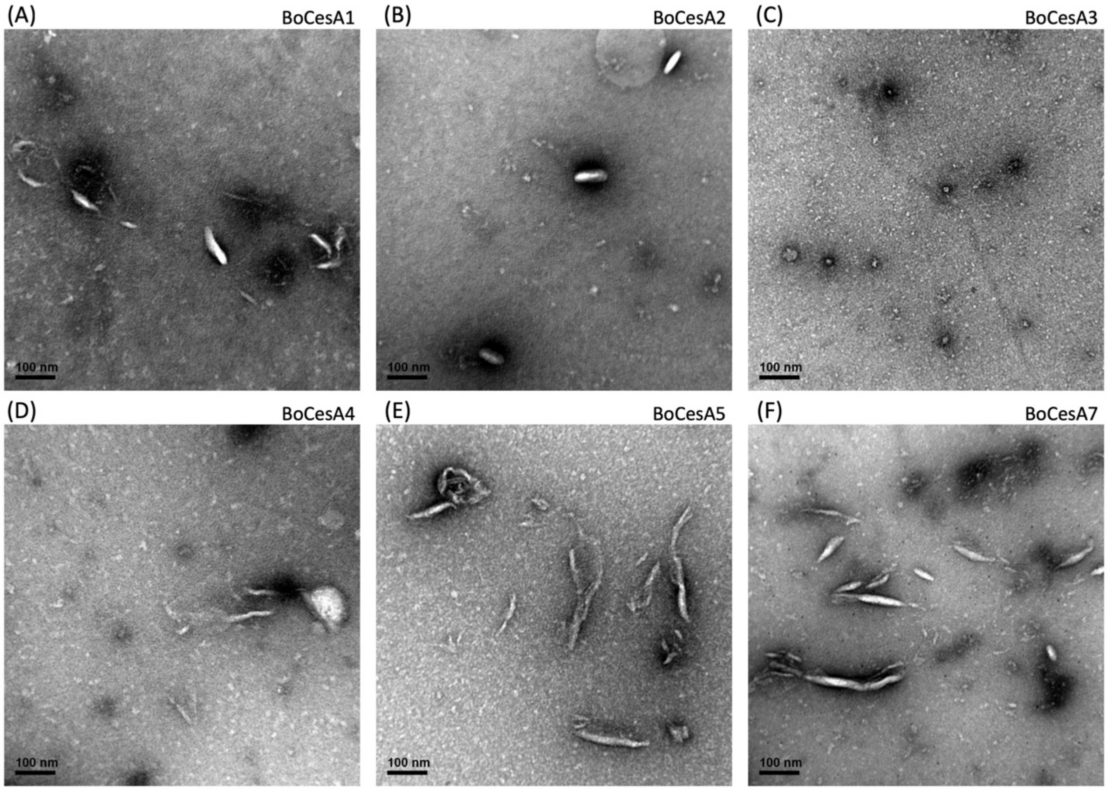

Figure S6. Negative staining of BoCesA synthesized product after cellulase treatment in TEM.

A-F: The synthesized product from BoCesA1, 2, 3, 4, 5, 7 with 15 hr cellulase treatment.
