# Supplementary material for: Heterologous overexpression, purification and functional analysis of plant cellulose synthase from green bamboo
Source: Plant Methods. 2019 Jul 25;15:80. doi: 10.1186/s13007-019-0466-0 (PMC6657065; doi:10.1186/s13007-019-0466-0)
Supplement: Supplementary file 7 — Additional file 7: Figure S7. GC–MS total ion chromatogram and mass spectrum of 1,4-glucan derivatives form n-Dodecyl-β-d-maltopyranoside (DDM). [file 13007_2019_466_MOESM7_ESM.pdf]

Figure S7

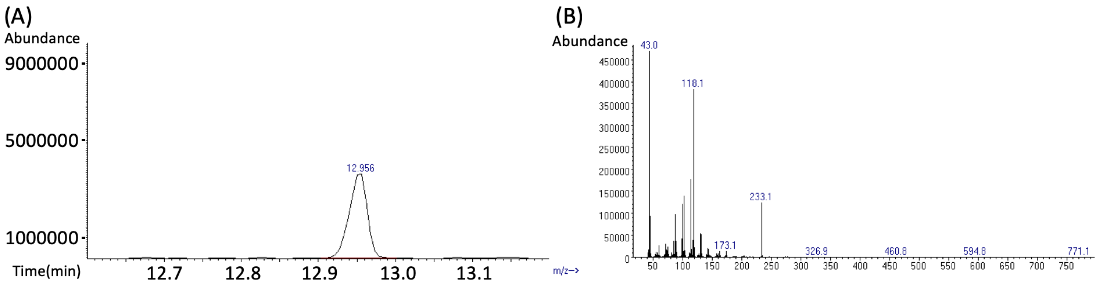

Figure S7. GC-MS total ion chromatogram and mass spectrum of 1,4-glucan derivatives form n-Dodecyl-β-D-maltopyranoside (DDM).

A: The peaks of 1,4-glucan derivatives contributed by n-Dodecyl-β-D-maltopyranoside.

B: The mass spectrum of 1,4-glucan derivatives peak in Additional file 7: Figure S7A.
